# Clinical presentation of pediatric patients with symptomatic SARS-CoV-2 infection during the first months of the COVID-19 pandemic in a single center in Mexico City

**DOI:** 10.3389/fped.2022.912784

**Published:** 2022-07-28

**Authors:** Ranferi Aragón-Nogales, Jessie Zurita-Cruz, Guillermo Vázquez-Rosales, Rafael Arias-Flores, Claudia Gómez-González, Victoria Montaño-Luna, Mariana Sámano-Aviña, Daniel Pacheco-Rosas, Eric Flores-Ruiz, Miguel Villasís-Keever, Guadalupe Miranda-Novales

**Affiliations:** ^1^Infectious Diseases Department, Pediatric Hospital National Medical Center XXI Century, Mexican Institute of Social Security, Mexico City, Mexico; ^2^Faculty of Medicine, National Autonomous University of Mexico, Pediatric Hospital Federico Gómez, Mexico City, Mexico; ^3^Hospital Epidemiology Division, Pediatric Hospital National Medical Center XXI Century, Mexican Institute of Social Security, Mexico City, Mexico; ^4^Analysis and Synthesis of the Evidence Research Unit, National Medical Center XXI Century, Mexican Institute of Social Security, Mexico City, Mexico

**Keywords:** COVID-19, SARS-CoV-2, children, adolescents, mortality, Latin America, morbidity, prognosis

## Abstract

**Background:**

The clinical spectrum of COVID-19 is broad, from asymptomatic to severe cases and death. The objective of this study is to analyze the clinical course of patients attended during the first months of the SARS-CoV-2 pandemic in a third-level pediatric hospital.

**Methods:**

*Design:* prospective cohort study. Patients with viral respiratory disease or suspected cases of COVID-19 were evaluated at the Pediatric Hospital, National Medical Center XXI Century, Mexico City, from 21 March 2020 to 13 January 2021. *Statistical analysis:* Chi-square test and Fisher’s exact test were used for comparisons; a logistic regression model was constructed to identify clinical or laboratory characteristics associated with critical disease. A *p*-value < 0.05 was considered statistically significant.

**Results:**

A total of 697 patients met the operational definition of viral respiratory disease or suspected cases of COVID-19 and underwent real-time reverse transcription polymerase chain reaction (rRT-PCR) SARS-CoV-2 testing. Patients with a positive result were included. Of the 181 patients (26%), 121 (66.8%) had mild disease and were treated as outpatients and 60 (33.1%) were hospitalized. A total of six patients met the criteria for multisystem inflammatory syndrome in children (MIS-C). Of the 60 inpatients, 65% were males, and 82% had one or more comorbidities. The main comorbidities were cancer (42%) and overweight (15%). The median hospital stay was 9 days. The inpatients had a higher frequency of fever, general malaise, dyspnea, chills, polypnea, and cyanosis than the outpatients (*p* < 0.05). Only 21.4% of the outpatients had one or more comorbidities, which were lower than in the hospitalized patients (*p* < 0.001). Laboratory data at admission were similar between critically ill and those with moderate and severe disease. The patients who developed pneumonia were at higher risk of critical disease, while older age was associated with a better prognosis. A total of 13 of the 60 inpatients died (mortality 7.1%). All but one had one or more comorbidities: four had cancer, four congenital heart disease, one chronic kidney disease and epilepsy, one Epstein–Barr virus-induced hemophagocytic lymphohistiocytosis, one obesity, and one diabetes mellitus.

**Conclusion:**

Hospital mortality is high, especially in children with comorbidities. Despite 2 years having passed since the beginning of the COVID-19 pandemic, the epidemiological and clinical data on children are still helpful to improve their prognosis.

## Introduction

The clinical spectrum of COVID-19 is broad, from asymptomatic cases to severe cases and death. Since the beginning of the pandemic, reports in the literature informed that children have a milder presentation and lower rates of hospitalization compared to adults. Severe cases and deaths are scarce (1).

Mexico is one of the countries most affected by the pandemic, according to data from the Ministry of Health as of 30 June 2022, 5.99 million cases had accumulated, with 326,000 deaths. In children (<18 years), 378,043 cases had been reported, 54.9% of them in adolescents who were 12–17 years old. A total of 1,276 deaths had been informed with a mortality rate of 0.33% (2).

The Directorate-General for Epidemiology established guidelines for epidemiological surveillance of viral respiratory disease (VRD). From the beginning and during the pandemic, the guidelines were modified to include COVID-19 cases. The definition of a suspected case is any person of any age who in the last 10 days has had one of the following signs and symptoms: cough, dyspnea, fever, or headache (in children under 5 years of age may be replaced by irritability) accompanied by at least one of the following: myalgias, arthralgias, sore throat, chills, chest pain, rhinorrhea, polypnea, anosmia, dysgeusia, and conjunctivitis. The process for notification to the National Epidemiological Surveillance System was established through the Electronic Platform for Epidemiological Surveillance System for Respiratory Diseases (SISVER for its acronym in Spanish) (3).

Given that there are few clinical-epidemiological publications that describe pediatric patients with COVID-19 in Mexico, the objective of this study is to analyze the clinical course of patients with confirmed symptomatic SARS-CoV-2 infection and to describe patients with multisystem inflammatory syndrome in children (MIS-C) attended during the first months of the COVID-19 pandemic in a third-level pediatric hospital in Mexico City.

## Materials and methods

### Patient cohort

A prospective study was conducted at the Pediatric Hospital, National Medical Center XXI Century, a tertiary care level hospital of the Mexican Social Security Institute (IMSS). During the pandemic, it was one of the three pediatric hospitals in Mexico City designated to provide care for COVID-19 patients. Before the conversion, the hospital had 184 beds and two intensive care units (neonatal and pediatric). With the emergency, a special in-patient unit with 6 beds and one area with 40 beds were enabled to receive patients with SARS-CoV-2 infection. Any patient classified as a suspected case of COVID-19, regardless of the health insurance status, was evaluated at the emergency department. The period of study was from 21 March 2020 to 13 January 2021.

Patients were included if met the operational definition of suspected VRD in accordance with the standardized guidelines for epidemiological and laboratory surveillance or were suspected cases of COVID-19 without respiratory symptoms. In both cases, only confirmed COVID-19 patients were analyzed, and no patient was excluded. The epidemiological and demographic data were provided by the primary caregiver, and a nasopharyngeal/oropharyngeal sample was taken for a real-time reverse transcription polymerase chain reaction test (rRT-PCR). According to the definitions of the guidelines and the test results, the patients were classified as follows:

(a) Negative case of COVID-19: a suspected case of VRD with a negative rRT-PCR test for SARS-CoV-2. (b) Positive case of COVID-19: a suspected case of VRD with a positive rRT-PCR test for SARS-CoV-2.

Also, patients referred from other hospitals with a positive rRT-PCR test were admitted directly to COVID units.

Epidemiological and demographic data were collected in the surveillance formats. Clinical records of hospitalized patients were reviewed for demographic information, underlying diseases, signs, and symptoms of infection, duration of disease, clinical course, and outcome.

Disease severity was classified as follows: (a) mild, in case of the presence of fever and/or asthenia and/or symptoms compatible with the upper respiratory infection without respiratory distress and/or instrumental evidence of pneumonia; (b) moderate, in the presence of respiratory distress and/or reduced nutrition and hydration and/or instrumental evidence of pneumonia; (c) severe, in the presence of severe respiratory distress and/or desaturation (SpO2 < 92% in ambient air) and/or intermittent cyanosis or apnea and/or systemic symptoms such as lethargy, dehydration, convulsions or suspected sepsis; and (d) critical, if acute respiratory distress syndrome (ARDS), multiorgan failure (MOF), septic shock, or coma occurs (4).

Also, patients were evaluated and classified as cases of MIS-C associated with COVID-19 according to the definition by the Centers for Disease Control and Prevention (5): an individual aged <21 years presenting with fever (>38.0°C for ≥24 h, or report of subjective fever lasting ≥24 h), laboratory evidence of inflammation [one or more of the following: an elevated C-reactive protein (CRP), erythrocyte sedimentation rate (ESR), fibrinogen, procalcitonin, D-dimer, ferritin, lactic acid dehydrogenase (LDH), or interleukin 6 (IL-6), elevated neutrophils, reduced lymphocytes and low albumin], and evidence of clinically severe illness requiring hospitalization, with multisystem (>2) organ involvement (cardiac, renal, respiratory, hematologic, gastrointestinal, dermatologic, or neurological); and no alternative plausible diagnoses; and positive for current or recent SARS-CoV-2 infection by rRT-PCR, serology, or antigen test; or COVID-19 exposure within 4 weeks prior to the onset of symptoms.

Follow-up of ambulatory patients was done daily by the hospital epidemiology staff by telephone calls, seeking for signs of alarm (fever >38.5 for more than 72 h, fast breathing, chest wall indrawing, inability to feed, or cyanosis) until resolution of signs and symptoms of the disease.

This study was approved by the Ethics Committee of the Pediatric Hospital and conducted in agreement with the ethical principles.

### Statistical analysis

Categorical data are presented as frequencies and percentages, and numerical data as the median and interquartile range (IQR) or minimum and maximum values, since they did not show normal distribution. Chi-square test, Fisher’s exact test, and Mann–Whitney U test were used to compare characteristics between groups. A logistic regression model was constructed to identify clinical or laboratory characteristics that differentiated patients with critical conditions from those with moderate and severe disease. A *p*-value < 0.05 was considered statistically significant. All the analyses were performed with SPSS version 24.

## Results

From 21 March 2020 to 13 January 2021, 697 patients with a median age of 8 years (1 day to 17 years) underwent rRT-PCR SARS-CoV-2 testing (nasopharyngeal/oropharyngeal sample). Gender distribution demonstrated male predominance with 385 patients (55.2%). Most were inhabitants of the Mexico City (*n* = 503, 72.1%), followed by the State of Mexico (*n* = 62, 8.8%), states of Morelos (*n* = 25, 3.5%), Chiapas (*n* = 19, 2.7%), and Guerrero (*n* = 19, 2.7%).

A total of one hundred eighty-one patients (26%) had a positive rRT-PCR SARS-CoV-2 test and 516 (74%) patients had a negative result. The frequency of positive cases was higher in the >12 year’s group with a statistically significant difference (*p* < 0.0001) in comparison with other age groups (Table 1).

**TABLE 1:** Age group and clinical characteristic of patients with a positive and negative RT-PCR SARS-CoV-2 test.

Age group/characteristic	RT-PCR positive test *n* = 181 (%)	RT-PCR negative test *n* = 516 (%)	Total *n* = 697 (%)	*p*-Value
1 day–5 years	58 (32)	207 (40)	265 (38)	<0.0001
6–12 years	47 (25.9)	170 (33)	217 (31.3)	
>12 years	76 (41.9)	139 (26.9)	215 (30.8)	
Fever	141 (77.9)	379 (73.4)	520 (74.6)	0.23
Headache/irritability*	103 (56.9)	245 (47.5)	348 (49.9)	0.029
Cough	98 (54.1)	228 (44.2)	326 (46.8)	0.021
Sore throat	73 (40.3)	117 (22.7)	190 (27.3)	<0.0001
Congestion or runny nose	65 (35.9)	155 (30)	220 (31.6)	0.14
General malaise	58 (32)	185 (35.9)	243 (34.9)	0.35
Dyspnea	55 (30.4)	125 (24.2)	180 (25.8)	0.10
Myalgias	51 (28.2)	124 (24)	175 (25.1)	0.26
Arthralgias	39 (21.5)	106 (20.5)	145 (20.8)	0.77
Abdominal pain	39 (21.5)	128 (24.8)	167 (24)	0.37
Thoracic pain	33 (18.2)	59 (11.4)	92 (13.2)	0.14
Chills	33 (18.2)	112 (21.7)	145 (20.8)	0.32
Diarrhea	32 (17.7)	123 (23.8)	155 (22.2)	0.08
Vomiting	29 (16)	124 (24)	153 (22)	0.02
Conjunctivitis	23 (12.7)	56 (10.9)	79 (11.3)	0.49
Polypnea	21 (11.6)	85 (16.5)	106 (15.2)	0.11
Anosmia**	13/123 (10.5)	6/309 (1.9)	19/432 (4.3)	<0.001
Dysgeusia**	10/123 (8.1)	8 (1.6)	18 (2.6)	0.12
Cyanosis	9 (5)	32 (6.2)	41 (5.9)	0.54

*In children under 5 years of age the sign was irritability and headache for 5 years and older.

**Only for children ≥6 years of age.

**FIGURE 1:**
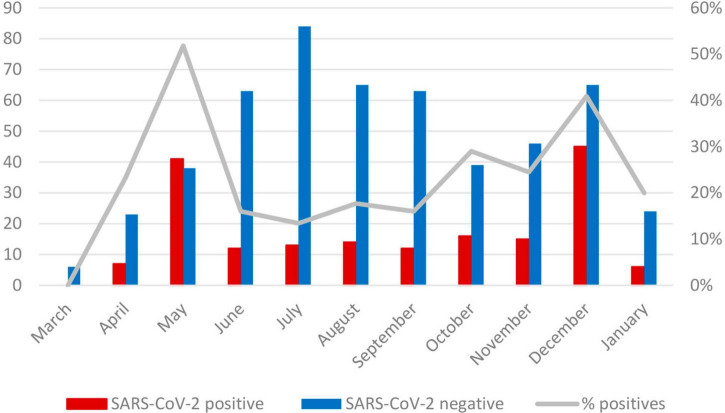
SARS-CoV-2 positive cases and positivity rate from 21 March 2020, to 13 January 2021, Pediatric Hospital National Medical Center, Mexico City.

Figure 1 describes the frequency of suspected cases with a symptomatic infection who were treated during the study period; as shown, the highest number of cases and positivity rate were in May and December 2020.

Table 1 compares the age and symptoms of patients with rRT-PCR SARS-CoV-2 positive and negative tests. Fever was the most common clinical manifestation, and headache/irritability, cough, sore throat, vomiting, anosmia, and dysgeusia more frequent among positive patients, which was statistically significant.

### SARS-CoV-2 positive cases

Of the 181 patients, 121 (66.8%) had a mild disease and were treated as outpatients, while 60 patients (33.1%) with a moderate, severe, or critical disease were hospitalized; 48/121 (26.5%) had epidemiological contact with a suspected or confirmed case. Of the outpatients, the median age was 10 years (range 2 months to 17 years); 66 were males (54.5%); 26/121 had some comorbidity (21.4%). The main comorbidities were lung/asthma disease in six patients (4.9%), cancer in 6 patients (4.9%), and overweight in 5 (4.1%).

Table 2 shows that the most common symptoms globally reported were fever, headache or irritability, cough, and sore throat. However, inpatients had a statistically significant higher frequency of fever, general malaise, dyspnea, chills, polypnea, and cyanosis in comparison with outpatients. Having sore throat and rhinorrhea were more frequent in outpatients.

**TABLE 2:** Clinical characteristics in patients with SARS-CoV-2 infection.

Initial clinical data	Inpatients *n* = 60 (%)	Outpatients *n* = 121 (%)	Total *n* = 181 (%)	*p*-Value
Fever	56 (93.3)	85 (70.2)	141 (77.9)	0.001
Headache/irritability*	32 (53.3)	71 (58.7)	103 (56.9)	0.49
Cough	28 (46.7)	70 (57.9)	98 (54.1)	0.15
Sore throat	15 (25)	58 (47.9)	73 (40.3)	0.003
Congestion or runny nose	10 (16.7)	55 (45.5)	65 (35.9)	<0.0001
General malaise	30 (50)	28 (23.1)	58 (32)	0.001
Dyspnea	25 (41.7)	30 (24.8)	55 (30.4)	0.02
Myalgias	15 (25)	36 (29.8)	51 (28.2)	0.50
Arthralgias	11 (18.3)	28 (23.1)	39 (21.5)	0.45
Abdominal pain	15 (25)	24 (19.8)	39 (21.5)	0.42
Thoracic pain	12 (20)	21 (17.4)	33 (18.2)	0.66
Chills	18 (30)	16 (12.4)	33 (18.2)	0.004
Diarrhea	15 (25)	17 (14)	32 (17.7)	0.06
Vomiting	12 (20)	17 (14)	29 (16)	0.30
Conjunctivitis	8 (13.3)	15 (12.4)	23 (12.7)	0.85
Polypnea	13 (21.7)	8 (6.6)	21 (11.6)	0.003
Anosmia**	2/42 (4.7)	11/108 (10.1)	13/150 (8.6)	0.28
Dysgeusia**	1/42 (2.3)	9/108 (8.3)	10/150 (6.6)	0.17
Cyanosis	7 (11.7)	2 (1.7)	9 (5)	0.007

*In children under 5 years of age the sign was irritability and headache for 5 years and older.

**Only for children ≥6 years of age.

Of the 60 inpatients, the median age was 9 years; 38 were males (64.4%); 49/60 patients had one or more comorbidities (81.6%) in comparison with outpatients (21.4%), and the difference was statistically significant (*p* < 0.0001). The main comorbidities were cancer in 25 patients (41.6%), followed by overweight in 9 patients (15%). A total of ten patients (16.6%) reported epidemiological contact with a suspected or confirmed case. The median hospital stay was 9 days.

### Inpatients’ clinical presentation and management

Of the 60 patients, 27 (45%) had a moderate illness, 16 (26.6%) had a severe illness, and 17 (28.3%) critical illness. Patients with severe and critical illnesses received intensive care. All critically ill patients except 2 had an underlying disease; 100% required mechanical ventilation support (median 4 days, range 1–22 days), 15 vasopressor support, 5 low-molecular-weight heparin, and 4 intravenous immunoglobulin (IVIG).

A total of thirty-one patients (16 with severe illness and 15 with moderate illness) received supplemental oxygen (including low-flow nasal cannula or face mask, high-flow nasal cannula, or non-invasive ventilation). All patients with supplemental oxygen received corticosteroids (48/60, 80%).

A total of fifteen critically ill patients received antimicrobial treatment due to a diagnosis of bacterial pneumonia or bacterial sepsis at admission (cefotaxime, ceftriaxone, cefuroxime, or cefepime), 11 cancer patients received piperacillin/tazobactam for an episode of fever and neutropenia, and 6 patients received antivirals during the first months of the pandemic (oseltamivir, acyclovir, and lopinavir/ritonavir). Three patients received cefotaxime plus metronidazole as they were diagnosed with acute appendicitis. Six patients had a healthcare-associated infection (ventilation-associated pneumonia, central line-associated bloodstream infection, and surgical site infection). Overall, 68.3% of the patients received antimicrobials during their hospital stay.

A total of six patients met the criteria for MIS-C. The median age was 9 years 5 months, and 3 of them had obesity. Four had a positive rRT-PCR test for SARS-CoV-2 infection, one had a confirmed household contact (mother), and one had a positive serology (IgG). The cardiac evaluation was normal in 4 patients; one patient had myocarditis (who also presented arrhythmia); and one pericardial effusion. All patients received IVIG and corticosteroids. One patient received a second dose of IVIG due to the persistence of fever and inflammation parameters (CRP and D-dimer). A total of four patients required supplemental oxygen and vasopressor support, and only one mechanical ventilation support. None of the six MIS-C patients died.

### Laboratory and imaging studies

Table 3 compares data from laboratory tests at admission between critically ill patients (*n* = 17) and those with moderate and severe disease (*n* = 43). As shown, the levels of hemoglobin, leukocytes, lymphocytes, neutrophils, and platelet counts were somewhat similar between the two groups.

**TABLE 3:** Comparison of laboratory data between critically ill patients and the group with moderate-severe COVID-19.

Variable	Critical disease *n* = 17	Moderate –severe disease *n* = 43	*p*-Value
		
	Median (IQR)	Median (IQR)	
Hemoglobin (g/L)	11.2 (8.7–12.9)	11.1 (8.3–14.8)	1.0
Leukocytes (cell/ml)	8,480 (5,675–17,660)	6,280 (1,690–10,190)	0.25
Lymphocytes (cell/ml)	1,400 (825–2,425)	1,150 (540–1,790)	0.25
Neutrophils (cell/ml)	6,100 (3,300–13,600)	3,500 (310–6,870)	0.25
Platelets (cell/ml)	172,000 (69,500–289,500)	137,000 (28,000–247,000)	0.56
C-reactive protein (mg/L)	29.2 (10.8–150.5)	25.1 (7.5–117.9)	0.73
Procalcitonin* (ng/ml)	0.80 (0.31–1.96)	0.34 (0.13–0.87)	0.18
D-dimer** (ng/ml)	693 (391.5–1,235.5)	552 (296.5–2,444.5)	0.24
Ferritin*** (ng/ml)	878.5 (146.7–2,334.7)	1,131 (496–2,078)	0.66

IQR, interquartile range.

*n = 15, n = 34, **n = 17, n = 41, ***n = 8, n = 18.

While CRP (median 29.2 vs. 25.3 mg/L), procalcitonin (median 0.80 vs. 0.34 ng/ml), and D-dimer (median 693 vs. 552 ng/ml) values were higher in the critical condition group, but the difference was not statistically significant (*p* > 0.05). The ferritin values were lower in the critically ill group (median 878.5 vs. 1,131 ng/ml), although only 36/60 patients had a result for this biomarker.

The laboratory results for the MIS-C patients showed a median hemoglobin value of 10 g/dl (IQR 9.4–14), leukocytes/mm^3^ median value 16,020 (IQR 7,367–26,005), neutrophils/mm^3^ median value 13,960 (IQR 6,090–233,375), lymphocytes/mm^3^ median value 955 (IQR 525–1,155), and platelets/mm^3^ median value 100,000 (IQR 46,500–261,000). A total of three patients presented acute renal injury with a median creatinine value of 1 mg/dl (IQR 0.57–4.41). All had elevated CRP levels, median 226 mg/L (IQR 122.5–361), and hypoalbuminemia, with a median albumin value of 2.2 g/L (IQR 1.84–2.46). Elevated D-dimer levels were observed with a median of 2,447 ng/ml (IQR 891–5,872) and fibrinogen values with a median of 138 mg/dl (IQR 363–577). Only three patients had ferritin reports with a median of 761 ng/ml (minimum 481 and maximum 761).

At admission, all hospitalized patients had a chest X-ray taken and in 13 it was reported as normal. The alterations associated with SARS-CoV-2 infection were bilateral interstitial infiltrates, micro and/or macronodular reticular infiltrates, pulmonary nodules, ground-glass opacities, air bronchogram, and crazy-paving pattern. Other abnormalities corresponded to the underlying disease or condition (cardiomegaly, pulmonary hypertension, lung metastases, pulmonary overdistention, and lymphadenopathy). Table 4 compares the radiological findings in detail according to the severity of COVID-19. Patients with alterations associated with the SARS-CoV-2 infection in chest X-rays had a higher risk of having a severe and critical illness in comparison with a normal study (40.7 vs. 6%), *p* = 0.001.

**TABLE 4:** Radiologic findings in 60 hospitalized patients according to clinical severity of COVID-19.

Chest X-ray finding	Moderate (*n* = 27)	Severe (*n* = 16)	Critical (*n* = 17)	Total*
Normal	11	1	1	13 (21.6)
Bilateral interstitial infiltrates	7	11	4	22 (36.6)
Micro-macronodular reticular infiltrates	12	5	3	20 (33.3)
Bilateral peripheral small opacities (nodules)	2	1	1	4 (6.6)
Glass-ground opacities	1	1	2	4 (6.6)
Air bronchogram	4	–	–	4 (6.6)
Crazy-paving pattern	1	–	–	1 (1.6)
Other findings**	4	2	2	8 (13.3)

*Several patients had more than one abnormality.

**Including: cardiomegaly, pulmonary hypertension, lung metastases, pulmonary overdistention, and lymphadenopathy.

A total of eight patients underwent CT scans, five reported images of bilateral ground-glass opacities, and areas of consolidation in two. In the remaining three, CT was requested for the underlying disease and no alterations secondary to the SARS-CoV-2 infection were reported.

### Unusual presentations

A total of five patients (8.3%) had an elevation of pancreatic enzymes three times the normal value, one was confirmed with Balthazar B pancreatitis by abdominal tomography, and in the rest, the imaging studies were inconclusive.

A total of four patients underwent exploratory laparotomy, three presented with acute abdomen, two were diagnosed with stage 4 appendicitis, and one with stage 1 appendicitis. The fourth patient had non-Hodgkin lymphoma and surgery was performed as part of the diagnostic approach.

A total of two infants (3.3%), 6 and 12 months of age, presented with encephalitis, one of them with a fatal outcome.

There was one obese patient with metabolic syndrome (1.6%) who presented with a thrombotic event at the hospital. At first, SARS-CoV-2 infection was not suspected. He denied previous respiratory or any infection symptoms. CT-angiography reported thrombosis of the popliteal artery and anterior and posterior tibial arteries, as well as popliteal vein thrombophlebitis. He received treatment with anticoagulants without improvement, so a supracondylar amputation was performed.

### Outcome

In the follow-up telephone calls, the staff collected information on the symptoms and overall status. None of the patients returned to the emergency department due to the worsening of the disease. Patients reported rapid improvement in a few days (3–5 days) and all of them recovered on the 10th day after the positive rRT-PCR test.

Table 5 shows the logistic regression model of clinical characteristics associated with a critical illness. Two variables were statistically significant: patients who developed pneumonia had a higher risk of critical condition (OR 9.879; 95% CI, 1.520–64.196), while older age was associated with a better prognosis (OR 0.987; 95% CI, 0.972–1.001). Abnormal chest radiograph findings had a high OR (9.071; 95% CI, 0.862–102.641); however, this was not statistically significant. Furthermore, in the logistic regression model, no laboratory data were associated with a critical COVID-19.

**TABLE 5:** Logistic regression analysis to determine factors associated to critical illness in hospitalized pediatric patients with COVID-19.

Variable	Critical illness *n* = 17	Moderate – severe illness *n* = 43	OR	95% Confidence interval	*p*-Value
	***n*** **(%)**	***n*** **(%)**			
Age (years)	4.1 (6 months–15 years)*	10 (2 months–16.5 years)*	0.987	0.972–1.001	0.05
Sex (female)	5 (29.4)	17 (39.5)	0.468	0.105–2.092	0.32
Malnutrition	13 (76.5)	29 (67.4)	1.123	0.192–6.572	0.89
Co-morbidity	15 (88.2)	33 (76.7)	4.417	0.528–36.922	0.17
Dyspnea	10 (58.8)	16 (37.2)	0.603	0.115–3.151	0.54
Diarrhea	8 (47.1)	10 (23.3)	1.529	0.298–7.833	0.61
Malaise	12 (70.6)	17 (39.5)	2.894	0.461–18.166	0.25
Abnormal chest X-ray findings	16 (94.1)	31 (72.1)	9.071	0.802–102.641	0.075
Pneumonia	14 (82.4)	18 (41.9)	9.879	1.520–64.196	0.016

*Median (minimum – maximum).

A total of 13 patients died (mortality 7.1%). All classified as having critical illness and requiring mechanical ventilation support. The median hospital stay of these patients was 12 days. All but one had comorbidities: four had cancer, four congenital heart disease, one chronic kidney disease and epilepsy, one Epstein–Barr virus-induced hemophagocytic lymphohistiocytosis, one obesity, and one diabetes mellitus. In 84.6% of the lethal cases, SARS-CoV-2 infection was among the conditions leading to the immediate cause of death. Table 6 enlists the causes and conditions leading to the fatal outcome.

**TABLE 6:** Cause-of-death according to the official certificate in hospitalized patients with SARS-CoV-2 infection.

Patient	Immediate cause of death	Conditions leading to the cause of death	Other significant conditions contributing to death
Male/14 years	Pulmonary hemorrhage	SARS-CoV-2 pneumonia	Osteosarcoma with lung metastasis
Female/2 years	Septic shock	(a) *Pseudomonas aeruginosa* bacteriemia (b) SARS-CoV-2 pneumonia	Cyanotic congenital heart disease, Down syndrome
Female/1 year	Acute renal failure	SARS-CoV-2 pneumonia	Epstein–Barr virus-induced hemophagocytic lymphohistiocytosis
Male/1 year	Acute respiratory distress syndrome	(a) SARS-CoV-2 pneumonia (b) Encephalitis	none
Male/6 years	Septic shock	Febrile neutropenia	Acute lymphoblastic leukemia SARS-CoV-2 infection
Male/9 years	Septicemia	(a) Acute respiratory distress syndrome (b) SARS-CoV-2 pneumonia	Non-Hodgkin lymphoma
Male/9 months	Cardiogenic shock	(a) Acute heart failure (b) Aortic coarctation	SARS-CoV-2 infection
Male/1 year	Cardiogenic shock	(a)Viral myocarditis (b) SARS-CoV-2 infection	Acyanotic congenital heart disease
Male/13 years	Septic shock	(a) Acute renal failure (b) COVID-19	Malignant brain tumor
Male/14 years	Acute respiratory distress syndrome	SARS-CoV-2 pneumonia	Chronic kidney disease, Epilepsy
Male/16 years	Acute respiratory distress syndrome	SARS-CoV-2 pneumonia	Obesity
Male/16 days	Acute respiratory distress syndrome	SARS-CoV-2 pneumonia	Cyanotic congenital heart disease
Female/16 years	Pulmonary hemorrhage	(a) Septic shock (b) SARS-CoV-2 pneumonia	Diabetic ketoacidosis

## Discussion

The behavior of a new virus cannot be anticipated until the first reports of the disease appear. At the beginning of the COVID-19 pandemic, it seemed that in the pediatric population, SARS-CoV-2 infection had a milder course, with fewer hospitalizations and low mortality compared to adults. In children, household contacts were the main source of contagion (6) also. It was assumed that, like other respiratory viruses, children would be responsible to maintain the chain of transmission in the community due to a higher rate of viral excretion and a greater number of asymptomatic cases (1). The estimates of SARS-CoV-2 infection were imprecise because children were not routinely evaluated. In a large series in China, infection was reported in 2.2% of the studied patients (7); in the United States, a retrospective cohort including 135,794 patients younger than 25 years, from 1 January through 8 September 2020, 4% were infected (8). In Mexico, a rate of 6.3% was reported in patients younger than 18 years until 26 June 2022 (2).

Of the total number of patients evaluated in this study, 181 (26%) had a positive rRT-PCR test. The percentage of positivity varied over time as the pandemic evolved in Mexico City. The demographic data coincide with the systematic review and meta-analysis by Liu et al. reporting 29 studies published from 12 December 2019 to 10 May 2020, including 4,300 children, with a higher proportion of cases in males, and scholars and adolescents (9). In another systematic review by Patel that included 2,832 patients from January to April 2020, COVID-19 was also reported more frequently in male patients 56.4% (10). In this study, percentage of the male gender was higher in hospitalized patients (64.4%), although it did not reach a statistically significant difference.

There are no distinctive clinical presentations of COVID-19 in children. The most common symptoms of positive cases in order of frequency were fever, headache or irritability, and cough, which is consistent with various publications (1, 8–10). These last two symptoms plus sore throat, dysgeusia, and anosmia were associated with having a positive result. Even though, dysgeusia and anosmia were referred in less than 6% of the cases. The symptoms associated with hospitalization were general malaise, dyspnea, chills, polypnea, and cyanosis. The clinical spectrum is broad but other symptoms occur less frequently, some less than 20% and there are few studies evaluating combinations of symptoms (11). Other authors had emphasized the presence of gastrointestinal signs and symptoms, but in our patients, abdominal pain and diarrhea presented with similar frequency in positive and negative cases. Gastrointestinal disorders include patients with acute appendicitis. In a study reporting acute abdomen and appendicitis in 1,010 pediatric patients with COVID-19 or MIS-C from Latin America, 38 children (3.8%) underwent abdominal surgery due to suspected appendicitis, 34 of them (89.7%) had an intraoperative diagnosis of acute appendicitis, and four of them had non-surgical findings (12). In this small cohort, three patients were confirmed with acute appendicitis. A retrospective cohort of 12,306 pediatric COVID-19 patients in the United States found a broad spectrum of non-specific symptoms across the age groups. Only 25.1% of the children had at least one of the typical symptoms (fever, cough, or shortness of breath), and 74.9% of the children did not have any of the typical COVID-19 symptoms (13).

According to the evidence in 15 contact-tracing studies (14), children and adolescents seem to have a lower susceptibility to SARS-CoV-2 infection than adults, with a pooled OR of 0.56 (95% CI, 0.37–0.85). Some reports have informed a high frequency of contact with a confirmed or suspected case of SARS-CoV-2 infection (11). In this cohort, the frequency was less than 30%. This low rate may have different explanations. COVID-19 increased the stigma against people suspected to have contact with COVID-19 patients (15). In the case of pediatric patients, parents were informed that children needed to be isolated, and visits would be restricted. Few were willing to accept this condition, so, they omitted contact information. Also, as most of the children had comorbidities, they continued visiting hospitals and clinics for medical attention, increasing the risk of exposition to SARS-CoV-2 during periods of high levels of community transmission.

Mild presentation of the disease (67%) and recognized alarm signs facilitated the follow-up program for ambulatory patients. As we did not continue the follow-up for a longer period, we lost the opportunity to identify patients with new or persistent symptoms or long-COVID cases. This entity, well recognized in adults, has been difficult to ascertain in children. In the meta-analysis by Behnood et al., there was a lack of a clear case definition, so the real prevalence of post-COVID-19 syndrome was not reported. Also, the primary analysis of controlled studies found that the frequency of the majority of reported persistent symptoms was similar in SARS-CoV-2 positive cases and controls (16). Workload and increasing acute cases demanding attention, as well as limited resources make it difficult to assess the long-term behavior in mild cases of COVID-19 in pediatric patients.

Published studies show that the immune response of children and adults to mild SARS-CoV-2 infection is similar but differs in severe diseases (17). The association of severe COVID-19 in children and adults with pre-existing medical conditions emphasizes the contributions of these comorbidities. The Pediatric Hospital is a tertiary care level specialty center and was one of the three pediatric hospitals designated to treat COVID-19 pediatric patients in Mexico City. Even though the hospital is for social security patients, during the pandemic any patient requesting care was received. In several studies, patients requiring hospitalization had a high frequency of comorbidities comparable to the report by Bustos-Cordova et al. from another tertiary care-level pediatric hospital in Mexico City—of the 50 patients with COVID-19, 52% had a previous medical condition (18). In a case series report from a second-level public hospital in Sinaloa, Mexico, during the first months of the pandemic (March–May 2020), only 10/51 confirmed cases were hospitalized, 50% had comorbidities, and one patient died (19). In our cohort, 81.6% of the inpatients had at least one comorbidity, higher than other reports. In the multicenter retrospective study of 1,063 children from 10 different Latin American countries, including Mexico, the presence of any comorbidity was associated with hospitalization (OR 5.3; 95% CI, 3.10–9.15), and the factors associated with ICU admission were residing in a rural area, metabolic or endocrine disorder, immune deficiency, and preterm birth (20). In contrast, in the present study, we could not verify that comorbidities were associated with critical illness, probably because 80% of the 60 hospitalized patients had an underlying disease. However, we determined that patients who developed pneumonia are at increased risk of a critical condition.

WHO’s interim guidance on the clinical management of COVID-19 published in May 2020 incorporates antibiotic stewardship principles with specific recommendations (21). The guidance does not recommend antibiotic therapy unless signs and symptoms of a bacterial infection exist, based on host factors and local epidemiology, along with daily assessments for de-escalation. At the beginning of the pandemic, little knowledge about the disease and a clinical presentation as severe pneumonia with elevated inflammatory biomarkers, led to an increase in antimicrobial use. Also, critical patients, with multiple invasive procedures and suboptimal practices of infection control and prevention, were associated with an increase in healthcare-associated infections. In our cohort, cancer patients with an episode of febrile neutropenia and COVID-19 received antimicrobial treatment according to the current guidelines. Also, 15/20 critical patients were suspected to have a bacterial infection, along with the patients with acute appendicitis and healthcare-associated infections, accounting for 68% of patients receiving at least one antibiotic. The use of antivirals occurred only in the first 4 months of the pandemic when scarce information was available. In comparison, in the systematic review that included 141 articles from 28 different countries, and 28,093 patients combined, 58.7% received antibiotics. In children it was 57% and in adults with comorbidity 74%. Most articles did not report bacterial coinfection, and more than 40 different antimicrobials were used to manage patients with COVID-19 disease (22). In a report from five Latin American countries (23), including 990 children with COVID-19 and MIS-C, the antibiotic prescription was 24.5%. The authors found that the rate of antibiotic prescriptions was significantly higher in children with MIS-C as well as those requiring respiratory support. Those with radiologic evidence of pneumonia/ARDS and those with fever, and the need for admission to the hospital were associated with a higher rate of antibiotic prescription. In our cohort, none of the ambulatory patients received antibiotics. If we consider a total of 181 patients with a positive rRT-PCR test, the antibiotic use was similar (22.6%).

In the Mexican population, the risk of death by age group and according to the presence of comorbidities has been analyzed in COVID-19 cases between 22 February 2020 and 18 April 2021. There were 52,432 total cases in Mexico and 52 deaths in the population group of those younger than 20 years in Mexico City. For the pediatric group, the case-fatality rate was below 0.3% (24), and the highest lethality was found in children <1 year with any comorbidity (5.8%). In our study, older patients had a lower risk of critical disease. This was similar to the findings of a retrospective cohort of 86 patients in another pediatric hospital in Mexico City, from 1 April to 28 September 2020, with four (5%) deaths: one previously healthy, two obese, and one a bone marrow transplant patient (25). In this report, mortality was greater (7.1%), and in most cases, SARS-CoV-2 infection was the condition leading to the cause of death. In the United States, COVID-NET reported, among 3,116 hospitalized children and adolescents with COVID-19 from 1 March 2020 to 19 June 2021, that only 21 (0.7%) died. And later, from 20 June to 31 July 2021, during the predominance of the delta variant, the mortality was higher, among 164 patients, three patients died (1.8%), but the difference between both periods was not statistically significant (26).

It is noteworthy that the hospital mortality reported here is higher than the national data, both for Mexico and the United States. This difference is most likely due to selection bias; pediatric patients in hospitals specifically dedicated to COVID-19 are likely to see more severe cases. Many of the patients were previously attended to our institution and most of them had more than one comorbidity. Of the 13 deaths, only one patient was without comorbidity and was 1 year old—a consistent risk factor in several studies (24, 26, 27). Very low mortality of 0.5% was reported in Argentina during the first year of the pandemic (28). In Brazil, in an analysis carried out approximately in the same period as the present study, the hospital mortality of pediatric patients was 7.6%, observing that there was a higher risk in children under 2 years of age, indigenous, and with some comorbidity (29). In a cohort of 557 severe and critical COVID-19 pediatric patients from North America, Latin America, and Europe, the overall hospital mortality was 10%, but 15% in children <2 years (OR 1.89; 95% CI, 1.05–3.39); 50% of the patients had comorbidity and 83% received antibiotics. Those with MIS-C had a lower mortality risk (OR 0.26; 95% CI, 0.11–0.64) (26) in contrast with other reviews from Latin America that found an overall case fatality ratio of 4% in 592 MIS-C cases (30). In a systematic review and meta-analysis that included 83 studies, 57 (21,549 patients) in the meta-analysis (of which 22 provided individual patient data) and 26 studies in the narrative synthesis, the authors found that hospitalized children and young people at the greatest vulnerability to severe disease or death with SARS-CoV-2 infection are infants, teenagers, those with cardiac or neurological conditions, or 2 or more comorbid conditions, and those who are significantly obese (31). In most of the reports from Latin America, these risk factors are common conditions.

This study has several limitations. The information on the outpatients is restricted to the data collected in the epidemiological report, and not all the comorbidities were registered as one option is “other” without specifying the disease. The follow-up of this group was finalized on the 10th day, so we could not assess the persistence of symptoms.

Inflammation biomarkers were not available for all the inpatients at admission; therefore, we could not determine if they could be considered prognostic factors. Chest CT was available for a limited number of patients, so we only compared findings in the chest X-rays. Lastly, the diverse comorbidities altered many of the laboratory values and imaging studies, making the comparison even more difficult.

## Conclusion

In this study, hospitalized COVID-19 pediatric patients had high mortality. Despite 2 years having passed since the beginning of the COVID-19 pandemic, the epidemiological and clinical data on children are still helpful to improve their prognosis.

## Data availability statement

The raw data supporting the conclusions of this article will be made available by the authors, upon reasonable request.

## Ethics statement

The studies involving human participants were reviewed and approved by the Local Ethics and Research Committee of the Pediatric Hospital Number 3603. Written informed consent from the participants’ legal guardian/next of kin was not required to participate in this study in accordance with the national legislation and the institutional requirements.

## Author contributions

RA-N, MV-K, and GM-N: study design, manuscript preparation, and final approval. RA-N, JZ-C, GV-R, RA-F, CG-G, VM-L, MS-A, DP-R, EF-R, and MV-K: collection and analysis, and interpretation of data. All authors contributed to the article and approved the submitted version.
